# Equine-Assisted Therapy for Posttraumatic Stress Disorder Among First Responders

**DOI:** 10.1177/00332941221146707

**Published:** 2022-12-21

**Authors:** Charles Nelson, Kimberly Dossett, Deanna L. Walker

**Affiliations:** Nelson Psychology Professional Corporation, Komoka, ON, Canada; Adjunct Professor, Department of Clinical Psychology, Western University, London, ON, Canada; Department of Clinical Psychology, Western University, London, ON, Canada; Nelson Psychology Professional Corporation, Komoka, ON, Canada; Department of Clinical Psychology, Western University, London, ON, Canada

**Keywords:** posttraumatic stress disorder, first responders, equine-assisted therapy

## Abstract

Equine-assisted therapy has emerged as an adjunctive integrative health modality in treating individuals experiencing physiological and psychological distress. However, limited research exists to assess the efficacy of such treatments as a possible adjunct to psychological treatment for Posttraumatic Stress Disorder (PTSD) in first responders. The current pilot study examines the additive benefits of equine-assisted exposure for first responders suffering occupational incapacitation from operational-related trauma. Seven first responders participated in an 8-week, 90-minute, equine-assisted therapy program. Primary outcome measures (i.e., anxiety, depression, trauma, inflexibility and avoidance) were administered pre- and post-intervention. Additional measures examined feelings about the self and views towards aspects of the program. Findings suggested initial support for symptom reduction, particularly for depressive and trauma-related symptoms. Qualitative feedback from participants suggested significant benefits including increased sense of peace, reduced anxiety, mindfulness, and increased trust in the self and others. To our knowledge, this is the first study to directly examine clinical outcomes of first responders with PTSD participating in equine-assisted therapy and presents a promising adjunct to care in first responders moving forward.

## Equine-Assisted Recovery from Posttraumatic Stress Disorder among First Responders

*“An insect crawls within reach of the lizard’s tongue and is gone. A herd of impala smell danger and move as one unit toward safety… animals immediately translate external clues into instinctual responses from within. Animal and environment are one, with no separation between stimulus and response.” -* Peter Levine (1997).

Dr. Levine’s (1997) seminal reflections on the natural and expected facility of trauma on shaping behaviour and experience illustrate the ever-present connection between humans and their environment: animals and humans both engineered to learn and discern danger from safety, inclusion from separation. Clinical psychologists integrating research with practice have seen the depths of human suffering and the bitter-sweet triumphs over extreme adversity. Decades of practice guidelines continue to refine and integrate best practices of recovery from Posttraumatic Stress Disorder (PTSD) including biological (pharmacotherapy), psychological (trauma-focused psychotherapy), as well as social reintegration and functional rehabilitation. Despite these appreciable and impactful advances in our understanding and treatment of PTSD, some thrive during recovery while others languish. Certainly, the pathway from exposure to trauma to growth from recovery is far more complex than iterative and aggregated practice guidelines can foretell.

Equine-assisted recovery from PTSD is one such adjunct to treatment, in addition to now standardized protocols for recovery, that shows tremendous promise in learning from animals that have evolved into experts in situational awareness, safety, and social (herd) advantages. The following pilot study examines the additive benefits of equine-assisted exposure to first responders suffering occupational incapacitation from operational-related trauma over treatment as usual.

### PTSD in First Responders

PTSD is a common and debilitating disorder among first responders. First responder occupations have historically included police officers, fire fighters, paramedics and search and rescue personnel (Haugen et al., 2012). These positions are characterized by routine exposure to both physical and psychological stressors including exposure to violence, severe injury, and death (Marchand et al., 2015; Marmar et al., 2006). Among the general Canadian population, there is an estimated 1.7% prevalence of PTSD (Statistics Canada, 2014; however, lifetime prevalence rates for high-risk groups, including military personnel, police, corrections workers, firefighters, and paramedics, range from 8% to 32% (Wilson et al., 2016). Long-term disabilities such as PTSD can be costly to both the individuals and their organizations in several dimensions due to healthcare costs, loss of productivity, loss of income and loss of economic growth due to capital and labour depletion (Trautmann et al., 2016). In 2010, mental and substance abuse disorders were the leading cause of years lived with disability and accounted for 10.4% of the global burden of disease (Wittchen et al., 2011). The importance of effective treatments and therapies for such disabilities are vital in protecting the value of life and protecting against potential costs to social and work environments.

### Treatment for PTSD

A substantial literature has examined the efficacy of various psychological interventions for PTSD, including cognitive processing therapy (CPT; Galovski et al., 2015), cognitive behavioural therapy (CBT; Kar, 2011), eye movement desensitization and reprocessing therapy (EMDR; Keenan & Royle, 2007) , as well as pharmacological interventions (Ipser & Stein, 2011). CPT is typically administered over 12 sessions, in individual and/or group formats, in which several phases of treatment are covered. These phases include assessment, education, processing, treatment, transition and relapse prevention (Galovski et al., 2015). Traditional CBT in the context of PTSD typically includes methods such as education, exposure and cognitive restructuring of core schemas and modification of cognitive distortions (Kar, 2011). However, very few studies have examined the efficacy of adjunct treatment strategies for PTSD among first responders, or for individuals in Canada (Lewis-Schroeder et al., 2019; Wilson et al., 2016).

### Equine-Assisted Therapy

Equine-assisted therapy is a form of experiential learning that has been developed in conjunction with licensed equine-assisted therapy instructors to offer horse-based learning activities to participants. As prey animals, horses tend to be sensitive to subtle changes in their environment and to the presence of humans (Burton et al., 2019). Horses are observed as mirroring and reflecting the emotions and energy of individuals who are in their presence and may offer feedback relating to an individual’s subtle nonverbal behaviour (Maros et al., 2008). They respond to both the behavior and the mood of individuals to whom they are exposed, allowing them to function as a biofeedback tool for those with whom they are interacting (Burton et al., 2019). In equine-assisted therapy, participants have the opportunity to gain insight into their own emotions and reactions through interactions with the horse (Masini, 2010). Activities often include grooming a horse, leading a horse, mindfulness/grounding exercises, facing obstacles with horses, round pen work with horses, observation of horses’ interaction with one another, and projection and processing of emotions.

Although equine-assisted therapy initially began as a treatment for physical disabilities in 1969 (Masini, 2010), recently there has been a growing use of equine-assisted therapies as an adjunctive integrative health modality in treating individuals who are experiencing a wide variety of mental health needs such as fear, depression, anger, anxiety, and autism (Burton et al., 2019; Masini, 2010). Though limited research exists to assess efficacy as an established psychological treatment, equine-assisted therapy has emerged as one possible adjunct to psychological treatment for PTSD as it allows participants to practice mindfulness, emotional regulation, and self-mastery or self-esteem building skills (Sylvia et al., 2020). Additional benefits may include an increased sense of well-being as a result of being in nature, increased feelings of acceptance, improvement in daily functioning, and increased awareness and connection with emotions (Masini, 2010; Shelef et al., 2019)). Preliminary evidence suggests that equine-assisted therapies may be helpful in reducing symptoms of PTSD in Veterans (Earles et al., 2015; Romaniuk et al., 2018; Shelef et al., 2019; Sylvia et al., 2020). To expand on these findings, it is essential to assess the efficacy of equine-assisted therapy for first responders given the primary symptoms of PTSD (i.e., hypervigilance, difficulty establishing a sense of meaning, and difficulty maintaining a positive self-image) (Sylvia et al., 2020).

### Present Study

Given the existing limited evidence regarding the use of equine-assisted therapy as an adjunct to treatment for first responders with PTSD, the current pilot study sought to evaluate preliminary evidence of the efficacy of equine-assisted therapy as a possible adjunctive treatment option for Canadian first responders with PTSD. We hypothesized that engagement in an eight-week equine-assisted therapy program would lead to significant reductions in anxiety, depressive, and trauma-related symptoms. As such, our research aims were as follows: 1) Quantitatively explore the association between participation in equine-assisted therapy as an adjunct to individual CPT treatment for PTSD, 2) Quantitatively monitor changes in symptomatology and other measures of well-being over the course of treatment, and, 3) Qualitatively explore perceived benefits of participating in equine-assisted therapy, outside of symptom reduction.

## Method

### Participants

Participants already involved in evidence-informed trauma-exposure based psychological treatment were recruited from a community psychology practice (Nelson Psychology Professional Corporation). Inclusion criteria for participants included: occupation as a first responder (e.g., police officer, paramedic, firefighter), fluent in English, pre-established diagnosis of PTSD, and currently on work-leave associated with traumatic adversity, but with current symptoms stable (non-acute, but with functional role impairment). Exclusion criteria included: allergies to horses, prior abuse towards animals, current significant substance abuse, active suicidal ideation, and psychosis.

Eight first responders consented to participate in the program, three of which were female. The age range was from 27 to 57 years with a mean age of 43.50 (*SD =* 10.41). Six of the participants were working with the police (regional, provincial, and national forces), one as a firefighter and one as a paramedic. One participant attended only a single session and had to subsequently drop out of the program due to scheduling conflicts. This participant was not included in calculations of results, resulting in a final sample of seven participants. Session attendance was moderate, with 25% of participants attending all eight sessions and 50% attending at least six of the eight sessions.

### Procedure

Pencil and paper versions of pre-treatment measures were completed by participants within two weeks prior to the start of their involvement in the equine-assisted therapy program. The equine-assisted therapy program consisted of eight weekly meetings of 90 minutes that took place at Belvoir Estate Farm (https://www.belvoirestatefarm.com) Meetings were facilitated by three licensed equine-assisted therapy instructors and a doctoral clinical psychology student (author K. D.). The content of the program was developed by licensed equine-assisted therapy instructors and was based on the Neuro-Equine Model developed by Hamilton and Hamilton (2019). Meetings covered material including grooming a horse, leading a horse, mindfulness/grounding exercises, facing obstacles with horses, round pen work with horses, and observation of horses’ interaction with one another. Participants learned about changes in the horse’s body language and expression and how the horses reflect the energy and emotion of humans and animals around them. Participants practiced describing how these cues changed in response to interaction with humans or other horses. Participants also learned to recognize their emotions beginning work with the horses, to change the intensity of their own energy, and to influence the behaviour of the horse. Pencil and paper versions of the same five post-treatment measures, as well as evaluation of the program components, were completed by participants (facilitated by author [*redacted]*) during the final meeting of the program.

### Measures

#### Primary Outcome Measures

##### Anxiety

To assess symptoms of anxiety, we administered the Generalized Anxiety Disorder-7 (GAD-7) questionnaire*.* The GAD-7 is a 7-item scale that measures the frequency of anxiety symptoms scoring from 0–21. The reliability and validity of the GAD-7 in terms of measuring general anxiety symptoms is good, and satisfactory with more specific disorders such as social phobia, or obsessive-compulsive disorder (Spitzer et al., 2006).

##### Depression

To assess depressive symptoms, we administered the Patient Health Questionnaire-9 (PHQ-9)*.* The PHQ-9 is a 9-item scale that measures depression symptoms frequency scoring from 0 “not at all bothered by the problem”, to 3 “bothered nearly every day” (Kroenke & Spitzer, 2002). The reliability and validity of the PHQ-9 in terms of measuring depression is good (Martin et al., 2006).

##### Trauma

To assess trauma-related symptoms, we administered the Post-Traumatic Stress Disorder Checklist for DSM-5 (PCL-5). The PCL-5 is a 20-item self-report measure designed to assess the DSM–5 symptoms of PTSD (Weathers et al., 2013). For each symptom, respondents provide a severity rating ranging from 0 “not at all”, to 4 “extremely”, that indicates the degree of distress associated with each symptom (Blevins et al., 2015).

##### Psychological Inflexibility and Avoidance

To assess psychological inflexibility and avoidance, we administered the Acceptance and Action Questionnaire-II (AAQ-II). The AAQ–II is a seven-item self-report measure that uses a 7-point Likert scale ranging from 1 “never true”, to 7 “always true” (Bond et al., 2011), whereby a higher score is indicative of higher inflexibility and avoidance. Sample items include “*I am afraid of my feelings*” and “*Emotions cause problems in my life*.” The AAQ–II exhibits a single-factor structure, good internal consistency, good test–retest reliability, and strong convergent validity with the original AAQ (Bond et al., 2011).

#### Study Specific Outcome Measures

##### Feelings Towards the Self

Two additional questionnaires were created specific to this study. The first questionnaire examined participants’ self-perceptions across five dimensions using a 7-point Likert scale from 1 “low”, to 7 “high”. Dimensions included self-esteem, self-confidence, self-acceptance, spiritual, and affiliative support. These dimensions were chosen based on the literature examining impacts of equine-assisted therapy (Gomez, 2017).

##### Usefulness of Program Parts

The second questionnaire was administered following the completion of the program and assessed participants perception of the usefulness of each of the six program components: time with horses, exposure to the labyrinth, natural surroundings, social support, staff, and involving loved ones. Participants rated these activities on a 7-point Likert scale from 1 “low”, to 7 “high”, and had the option of adding comments about their experiences with each of the activities. Open-ended responses were analysed for key themes and primary takeaways regarding the program experience. In addition to these data, researchers and instructors made notes of behavioural observations throughout the course of the program.

### Data Analysis

To describe the characteristics of the sample, we computed frequency distributions, means and standard deviations for continuous variables and calculated percentages for categorical variables. For the primary outcome measures, we performed paired t-tests to examine the effects of the intervention from pre-test to post-test. As study-specific measures were not standardized and were exploratory in nature, we performed a paired t-test to assess pre-test to post-test for feelings towards the self and have described additional findings in text. Qualitative data were analyzed to explore present themes relating to participants’ experiences. In addition to these data, researcher and instructor observations are described herein.

## Results

### Primary Outcome Measures

The mean scores and standard deviations for all primary outcome measures, pre- and post-intervention are presented in Table 1. Results will be reviewed for each measure in turn.

**Table 1.** Pre-and Post-Intervention Scores on Primary Outcome Measures.

Outcome	Total Score		t-test	Clinical Cut-off		DSM-5 Criteria	
	Pre-Intervention M (SD)	Post-Intervention M (SD)	*t* (*p)*	Pre-Intervention	Post-Intervention	Pre-Intervention	Post-Intervention
Anxiety symptoms	10.57 (5.68)	7.71 (5.47)	1.29 (0.24)	3	3	N/A	N/A
Depressive symptoms	14.21 (8.57)	9.28 (6.85)	2.52 (0.46)	N/A	N/A	4	2
Trauma-related symptoms	46.29 (18.33)	28.28 (19.75)	4.09 (0.01)	5	3	5	3
Inflexibility and avoidance	40.00 (9.57)	38.07 (7.74)	0.86 (0.42)	N/A	N/A	N/A	N/A

Note. *N* = 7; The Acceptance and Action Questionnaire – II is scored such that a lower total score indicates increased flexibility and decreased avoidance, while a higher total score indicates less flexibility and more avoidance; Clinical cut-off refers to the number of participants above the cut-off score for the anxiety and trauma symptom measures; DSM-5 Criteria refers to the number of participants meeting criteria for DSM-5 diagnoses based on questionnaire responses.

#### Anxiety Symptoms

Although there was no statistically significant difference between pre- and post-intervention scores, five of the seven participants indicated a reduction in anxiety symptoms from pre-to post-intervention. Similarly, five met criteria for a lower severity of anxiety symptoms following the intervention.

#### Depressive Symptoms

A statistically significant difference between pre- (*M* = 14.21, *SD* = 8.57) and post-intervention (*M* = 9.28, *SD* = 6.85) scores indicated a reduction in symptoms of depression, *t* (6) = 2.5, *p* < 0.05. Pre-intervention, four participants met criteria for depression according to scoring of the PHQ-9 (based on the DSM-5 criteria). Following intervention, only two of these participants continued to meet criteria for depression.

#### Trauma-Related Symptoms

There was a statistically significant difference between pre- (*M* = 46.28, *SD =* 18.33) and post- (*M* = 28.28, *SD* = 19.75) intervention scores, *t* (6) = 4.09, *p* < 0.01. Further, pre-intervention five participants were above the clinical cut-off on the PCL-5, and met criteria for PTSD according to the DSM-5 criteria, whereas only three of these participants still met these specifications post-intervention

#### Psychological Inflexibility and Avoidance

No statistically significant difference was found between pre- and post-intervention scores on the AAQ-II, however five of the participant’s scores indicated increased flexibility (i.e., reduced scores) from pre-to post-intervention.

### Study-Specific Outcome Measures

#### Feelings Towards the Self

All but one participant endorsed feeling more positively about themselves post-intervention compared to pre-intervention. Results of paired t-test suggested a significant increase in positive feelings towards the self from pre-test (*M =* 20.93, *SD =* 7.11) to post-test *(M =* 24.50, *SD =* 8.42; (*t* (6) = 3.09, *p* = .02). For individual scores, the greatest change was seen in affiliative support, where participants endorsed a score at post-intervention that was 1.35 points higher than pre-intervention. This may have been influenced by social support from other participants as well as staff. Four participants noted an increase of one point in self-esteem, and two noted an increase of one point in self-confidence. Five participants endorsed an increase of one or more points in their spiritual interest and six endorsed an increase in affiliative support from pre-to post-intervention. Three participants endorsed increases of one or more points in self-acceptance, while two participants noted decreases in self-acceptance post-intervention.

#### Usefulness of Program Parts

All components of the program were rated at least 6.3 out of seven following participants’ completion of the program, suggesting overall satisfaction with the program. Participants rated time with the horses as the most helpful, followed by staff. Notably, participants consistently mentioned that time with the horses brought them feelings of calm and helped them to be present and mindful in the moment (see Table 2 for example qualitative responses). Participants also reported experiencing increased trust in themselves and in others, and a more positive outlook on life. Most participants reported feeling a sense of belonging and understanding in participating in the program with other first responders experiencing PTSD; however, one first responder noted that this was also a difficult component of the program. Overall, participants noted that staff were knowledgeable, friendly, and understanding.

**Table 2.** Qualitative Responses for Feedback relating to Program Elements.

Theme	Example Quotes
Feeling of peace, calm, clear mind, reduced anxiety	“Had a feeling of calm during and immediately after spending time with the horses, also a feeling of having a clear mind and a better outlook on life in general” “Similar feeling during the time spent with the horses, peace and quiet, the sound of other horses in their stalls, wildlife outside the barn” “Very peaceful and calm” “There were days when I attended that my anxiety and anger was high. Working with horses grounded my feelings and I was able to focus and manage my feelings” “The actual time working with/touching the horses are some of the very few times where I felt some “peace” over the past 8 weeks”
Mindfulness	“Being present with the horses allowed me to focus on leading and being present with the horses. Allowed me to not think about the issues of the day” “Lots of beauty and energy on the farm” “I find no happiness in urban environments and need connections with nature in order to be a “good” person” “Connecting to the horses, they seem to be able to look directly into your soul, they see and feel your pain/emotions” “Extremely effective tool to check in with the real feelings inside. Instead of the fake façade we portray. Really being honest with yourself, brining yourself to the present to work with the horses is an incredible tool.” “Brings such peace. Gives a good surrounding to “check out” of our stressful exterior world and fully “check in” with nature and our surroundings”
Increased trust in others	“When I first attended I felt as though I couldn’t trust the others having not known them or their backgrounds. That quickly changed, most notably during the exercises with the horses in the group setting when attempting the given task”
Increased trust in self	“Spending direct time with the horses helped me control my anxiety and helped me with trusting myself”
Sharing with others of similar experiences	“It was great spending time with first responders that also have PTSD and their own journey and understand my journey” “Was great sharing feelings allowed us to reflect with our own personal journey and understand we are not alone” “There is always a sense of companionship spending time with first responders who battle with PTSD” “Common understanding, no judgement” “Although not much talking amongst us happened, it felt great to be part of a group you know understands what you’re going through” “I found being around the other first responders to be the most difficult part of the program”
Positive experiences with staff	“The staff provided a feeling of welcomeness and belonging, also a feeling of understanding of our circumstances” “The staff was amazing, friendly, and understanding” “Very knowledgeable and patient and did an amazing job explaining time with horses” “Great staff and very understanding” “The staff were all very welcoming and supportive and positive individuals” “Good staff, understanding, compassion, love” “They were great. Treated us as we were friends, teaching in a kind and compassionate way. They were all so knowledgeable but delivered the content in a digestible way”

### Instructor Observations

Further to qualitative feedback from participants, instructors and the researchers took memo notes relating to the benefits that they observed throughout the program. In addition to participant feedback, staff noted improvements in participants’ confidence working around the horses throughout the program. For example, in the first session, only two participants were willing to work in the round pen with a loose horse, whereas all were enthusiastic to do so by the final session. This mirrors improvements noted by participants in their self-esteem and self-confidence.

From the first session, participants were observed encouraging one another and cheering on each other’s successes. Over the course of the program, participants were observed speaking more openly during group check-ins about their reactions to sessions and emotional experiences both in-session and during the time between sessions. Participant’s responses to the open-ended questionnaire supported this observation, with numerous participants noting the importance of companionship and understanding from other participants.

## Discussion

Overall, the results of this pilot project suggest the use of equine-assisted therapy as an adjunct to evidence informed trauma exposure psychological treatment provided benefit to first responses with PTSD in terms of clinical symptomatology. In line with our predictions, participants experienced significantly lower levels of depression and trauma-related symptoms following completion of the program. Notably, numerous participants who had met threshold for depression and PTSD prior to the beginning of the program no longer met criteria following their participation in the program. There was some preliminary evidence to suggest decreases in anxiety and inflexibility/avoidance over the course of the program; however, further data are needed to examine the extent of this relationship.

In addition to quantitative findings, qualitative reports suggest positive experiences for participants who engaged with this program. Participants reported experiencing reduced anxiety and an increased ability to be mindful in the moment. The program was consistently associated with a sense of calm, peace, grounding, and sense of being able to trust self and others. This is particularly meaningful given primary concerns associated with PTSD-symptoms relating to reduced sense of trust in others and the self. Connecting with oneself, as well as with others who have shared experiences, appeared to greatly benefit first responders. These findings align with previous research in this area to suggest the benefits of creating a safe space for individuals experiencing mental distress to connect with their feelings, understand how their actions and emotions affect themselves and others, and practice coping and problem-solving skills (Masini, 2010; Moore et al., 2009). These findings also support the existing literature which suggests the benefits of equine-assisted therapy in improving self-esteem and self-mastery (Bachi, 2013; Earles et al., 2015; Ferruolo, 2016).

### Limitations

Given the pilot nature of the current study, our initial sample was limited in size. With promising initial findings and feedback from participants, we recommend future investigation of this potential adjunct to treatment within the structured context of a larger clinical trial. Similarly, given that all participants were also concurrently involved in psychological treatment for PTSD, further research could seek to examine the efficacy of equine-assisted therapy as a primary treatment for PTSD (e.g., with and without additional concurrent treatment, psychopharmacological intervention, and control group). Larger sampling should also seek to examine differences in treatment involvement and response across gender and race, as well as those with multiple comorbidities. Similarly, with early research in equine-assisted therapy for children suggesting enduring effects of reduced distress at a six-month follow-up (Klontz et al., 2007; Romaniuk et al., 2018), it would be beneficial to examine longitudinal effects of this treatment following the conclusion of the program.

Finally, it is notable that one participant reported that participating alongside other first responders was a challenge for them. Although research has suggested an overwhelming positive experience of participants who engage in group treatment for PTSD, particularly relating to increased social contact, normalized experiences and symptoms, and commitment to treatment (Sloan et al., 2012), it is still reasonable that some participants may not feel comfortable engaging in a group therapy setting. Similarly, scheduling conflicts interfered with one participant’s involvement in the study. This is to be expected given the shifting nature of first responders’ work schedules. As a result, we recommend clinical judgment in assessing individual patients’ readiness for change and level of comfort in social settings prior to engaging in group treatment such as equine-assisted therapy, as well as the nature of their schedule that may interfere with adherence to weekly sessions.

## Conclusion

The current pilot project is, to our knowledge, the first to examine the use of equine-therapy as an adjunct to evidence-informed trauma-exposure based psychological treatment for first responders with PTSD. Emerging findings provide important information about the perceived benefits of equine assisted therapy for Canadian first responders and add to the growing literature examining the effectiveness of equine-assisted therapy for PTSD. Our hope is that the benefits experienced by first responders in this pilot project prompt larger scale research in this domain, whereby this program could be offered to a wider group of Canadian first responders who are experiencing mental distress, including anxiety, depression, and PTSD.
